# Genetic Variants Associated with Arsenic Susceptibility: Study of Purine Nucleoside Phosphorylase, Arsenic (+3) Methyltransferase, and Glutathione *S*-Transferase Omega Genes

**DOI:** 10.1289/ehp.10581

**Published:** 2008-01-14

**Authors:** Sujata De Chaudhuri, Pritha Ghosh, Nilendu Sarma, Papiya Majumdar, Tanmoy Jyoti Sau, Santanu Basu, Susanta Roychoudhury, Kunal Ray, Ashok K. Giri

**Affiliations:** 1 Molecular and Human Genetics Division, Indian Institute of Chemical Biology, Kolkata, India; 2 Nil Ratan Sircar Medical College and Hospital, Kolkata, India; 3 Peerless Hospital and B.K. Roy Research Centre, Kolkata, India; 4 Department of Medicine, Calcutta National Medical College, Kolkata, India; 5 Department of General Medicine, Sri Aurobindo Seva Kendra, Kolkata, India

**Keywords:** arsenic, *As3MT*, *GSTO1*, *GSTO2*, *PNP*, skin lesion, susceptibility

## Abstract

**Background:**

Individual variability in arsenic metabolism may underlie individual susceptibility toward arsenic-induced skin lesions and skin cancer. Metabolism of arsenic proceeds through sequential reduction and oxidative methylation being mediated by the following genes: purine nucleoside phosphorylase (*PNP*), arsenic (+3) methyltransferase (*As3MT*), glutathione *S*-transferase omega 1 (*GSTO1*), and omega 2 (*GSTO2*). *PNP* functions as arsenate reductase; *As3MT* methylates inorganic arsenic and its metabolites; and both *GSTO1* and *GSTO2* reduce the metabolites. Alteration in functions of these gene products may lead to arsenic-specific disease manifestations.

**Objectives:**

To find any probable association between arsenicism and the exonic single nucleotide polymorphisms (SNPs) of the above-mentioned arsenic-metabolizing genes, we screened all the exons in those genes in an arsenic-exposed population.

**Methods:**

Using polymerase chain reaction restriction fragment length polymorphism analysis, we screened the exons in 25 cases (individuals with arsenic-induced skin lesions) and 25 controls (individuals without arsenic-induced skin lesions), both groups drinking similar arsenic-contaminated water. The exonic SNPs identified were further genotyped in a total of 428 genetically unrelated individuals (229 cases and 199 controls) for association study.

**Results:**

Among four candidate genes, *PNP, As3MT, GSTO1,* and *GSTO2*, we found that distribution of three exonic polymorphisms, His20His, Gly51Ser, and Pro57Pro of *PNP*, was associated with arsenicism. Genotypes having the minor alleles were significantly overrepresented in the case group: odds ratio (OR) = 1.69 [95% confidence interval (CI), 1.08–2.66] for His20His; OR = 1.66 [95% CI, 1.04–2.64] for Gly51Ser; and OR = 1.67 [95% CI, 1.05–2.66] for Pro57Pro.

**Conclusions:**

The results indicate that the three *PNP* variants render individuals susceptible toward developing arsenic-induced skin lesions.

Arsenic is recognized as a potent environmental toxicant that causes severe health problems in populations chronically exposed to arsenic-contaminated drinking water. However, the disease manifestations often depend on individual genetic variability. Corroborative of this view, although more than 6 million individuals in West Bengal, India, are endemically exposed to inorganic arsenic, only 300,000 people show arsenic-induced skin lesions, the hallmark sign of chronic arsenic exposure (Chakraborti et al. 2002). This fact clearly elucidates that genetic variability plays a critical role in susceptibility toward arsenic toxicity. It is worthwhile to mention that in West Bengal, groundwater in 9 of 18 districts is contaminated with arsenic, far above the acceptable limit of 10 μg/L [Frost et al. 2003; World Health Organization (WHO) 2004]. Chronic arsenic exposure causes various skin manifestations that include keratosis on palms and soles, hypopigmentation, characteristic raindrop pigmentations on chest, back, and legs, and in extreme cases, *in situ* carcinoma or Bowen disease (Basu et al. 2004; Guha Mazumder 2003). These skin lesions generally develop with a latency period spanning more than 10 years from first exposure; however, the latency period may be as short as 6 months, depending on the concentration of arsenic in drinking water, volume of intake, and health and nutritional status (Haque et al. 2003). In addition to skin lesions, other clinical manifestations of chronic arsenicism include peripheral neuropathy, peripheral vascular diseases, respiratory problems, conjunctivitis, various reproductive abnormalities, and ultimately, malignancies in a number of organs including skin, lung, and bladder [International Agency for Research on Cancer (IARC) 2004]. Although it is well known that arsenic can cause cellular toxicity and carcinogenicity, the underlying mechanism is yet undefined. Our survey of the published literature (1992–2007) found that alterations in the arsenic metabolism pathway, recently termed “arsenic biotransformation,” may actually explain the molecular mechanism of arsenicism.

To date, two pathways have been proposed to elucidate arsenic metabolism in humans. The classical pathway suggests that once arsenate (As^V^) enters the cell via phosphate transporter [National Research Council (NRC) 1999], it undergoes sequential reduction and oxidative methylation, with only one end product, dimethyl arsinate (DMA^V^) (Vahter 2002). The newly proposed alternative pathway suggests that arsenic either binds to certain proteins (Naranmandura et al. 2006) or conjugates with glutathione (Hayakawa et al. 2005), and subsequent methylation results into two end products: methyl arsonate (MMA^V^) and DMA^V^. In both pathways, purine nucleoside phosphorylase (PNP) reduces As^V^, and glutathione *S*-transferase omega 1 (GSTO1) and omega 2 (GSTO2) reduce all the pentavalent arsenic species (As^V^, MMA^V^, and DMA^V^). Conversely, arsenic (+3) methyltransferase (As3MT) methylates the trivalent arsenic species (As^III^, MMA^III^, and DMA^III^) (Engström et al. 2007).

Given the roles of these enzymes in arsenic biotransformation, functionally relevant genetic polymorphisms of these genes (*PNP*, *As3MT*, *GSTO1*, and *GSTO2*) are likely to produce interindividual variations in arsenic metabolism and thus susceptibility toward arsenic toxicity. To identify the genetic variants, we performed detailed screening of exonic regions in arsenic-metabolizing genes. Information regarding all genes studied was obtained from our review of the Single Nucleotide Polymorphism database (http://www.ncbi.nlm.nih.gov/sites/entrez) and published literature, which suggests that six polymorphisms occur in the exonic region of *PNP* gene, containing six exons (accession no. NM_000270, location 14q11.2). Among these, one is located in the 5′UTR (rs17881206); three (His20His, Gly51Ser, Pro57Pro) in exon 2; one in exon 5 (Ala174Ala); and one in the 3′UTR (rs7785) (Yu et al. 2003). Several exonic single nucleotide polymorphisms (SNPs) are also reported (Arg173Trp, Met287Thr, Thr306Ile, Ile132Phe, Tyr135Asn, Gly140Ala) (Wood et al. 2006) in *As3MT* containing 11 exons (NM_020682, location 10q24). Two members from the glutathione *S*-transferase family, *GSTO1* (NM_004832) and *GSTO2* (NM_183239), mapped to chromosome 10q are 7.5 kb from each other and contain 6 exons each. In *GSTO1*, although six exonic SNPs (Cys32Tyr, Ala140Asp, −/AGG, Glu208Lys, Thr217Asn, Ala236Val) have been identified in various world populations (Mukherjee et al. 2006), there has been a single report of a Thr217Asn variant in the dbSNP but not yet reported in any population (Whitebread et al. 2003). Four nonsynonymous exonic SNPs (Val114Ile, Cys130Tyr, Asn142Asp, Leu158Ile) were also reported in *GSTO2* (Mukherjee et al. 2006).

Our goal in this study was to identify exonic SNPs in the above-mentioned arsenic-metabolizing genes in the arsenic-exposed population of West Bengal and to ascertain a possible association between any of the identified exonic SNPs and development of arsenic-induced skin lesions.

## Materials and Methods

### Study sites and subject selection

From previous survey reports, we selected three districts of West Bengal that were identified as the most arsenic-affected region at the level of ground-water contamination (Chowdhury et al. 2000). For our field survey, we chose several villages in the following three districts: North 24 Parganas (five villages from four administrative blocks designated as Gaighata, Habra, Deganga, and Baduria), Nadia (two villages from Haringhata block), and Murshidabad (three villages from Bhagabangola block I and Bhagabangola block II). The detailed procedure of field survey and sample collection has been described previously (De Chaudhuri et al. 2006; Ghosh et al. 2006, 2007). In the present study, we recruited a total of 428 genetically unrelated study subjects, including 229 cases and 199 controls. The inclusion criteria for cases were based on the presence of more than one characteristic skin lesion, the hallmark sign of arsenicism as diagnosed by dermatologists. For selection of controls, we recruited genetically unrelated individuals without arsenic-induced skin lesions from the same villages; preferentially, family members of the cases who were related by marriage so that the exposure level through drinking water was similar. During our epidemiologic survey, we carried out a detailed pedigree analysis for each proband, rejecting the selection of parent–offspring or siblings from the same family, to avoid genetic overmatching. We also took the detailed case reports of study participants, including age, sex, addiction (in the form of both tobacco smoking and chewing), occupation, food habits, source of drinking water, and medical history. Questionnaire-generated data revealed that these individuals were exposed to arsenic only through groundwater, as tube wells were the only source of drinking water in these villages; any possibility of arsenic consumption through seafood was also ruled out. We obtained informed consent from all the study participants prior to the collection of drinking water and other biological samples such as blood, urine, nail, and hair. The study protocols adhered to the tenets of the Declaration of Helsinki II (2000) and was approved by the Indian Institute of Chemical Biology Institutional Review Board.

### Arsenic exposure assessment

To assess the overall load of arsenic in the body, we estimated arsenic content in drinking water, nail, hair, and urine samples. As arsenic shows strong reactivity and therefore affinity toward thiol compounds, it preferentially accumulates in peripheral keratin-rich tissues such as skin, nail, and hair. The detailed description of arsenic estimation and pretreatment procedure of biological samples and water has been reported previously (Buchet et al. 1981; Chatterjee et al. 1995; Das et al. 1995). We used flow injection–hydride generation–atomic absorption spectrometry (FI-HG-AAS) to estimate arsenic content in different biological samples (urine, nail, and hair) and drinking water. A Model Analyst-700 spectrometer at our institute equipped with a Hewlett-Packard Vectra computer with GEM software (Hewlett-Packard, Houston, TX, USA) , and arsenic lamp (lamp current 380 mA) was used for this purpose.

### Genotyping

Exon screening for all the genes was initially carried out in 25 cases and 25 controls to identify the exonic SNPs in our population. All identified polymorphisms in our population were further validated in 229 cases and 199 controls for association study. DNA extraction from blood was carried out using standard protocols (Sambrook 1989). To amplify the exonic regions, polymerase chain reaction (PCR) was performed in a 25-μL reaction volume using standard buffer, MgCl_2_ (1.5 μM), deoxyribonucleotides (200 μM), and Taq polymerase supplied by Life Technologies, Carlsbad, CA, USA) in an MJ Research PTL-225 thermocycler (GeneAmp-9700; Applied Biosystems, Foster City, CA, USA). The sequences of flanking primers (Clontec, Mountain View, CA, USA) for exons of all the genes are described in Supplemental Material, Table 1 (online at http://www.ehponline.org/members/2008/10581/suppl.pdf). All PCR products were analyzed by agarose (1.5%) or polyacrylamide gel (6%) electrophoresis, stained with ethidium bromide, and photographed under ultraviolet light. Bidirectional sequencing was done in an ABI prism 3100 DNA sequencer (Applied Biosystems), using Big Dye Terminator, pre-treated with Exo-SAP (Amersham Life Sciences, Little Chalfont, Buckinghamshire, UK). Samples with ambiguous chromatograms, as well as those containing SNPs observed in only a single sample, were subjected to a second, independent round of amplification, followed by DNA sequencing. The sequence chromatograms obtained were analyzed with Chromas 2.32 (Technelysium Pty Ltd, Tewantin, Australia) and compared with the reference sequence for genotyping of respective amplicons [Supplemental Material Table 2 (online at http://www.ehponline.org/members/2008/10581/suppl.pdf)]. The single exonic SNP found in *GSTO2* (Asn142Asp) was genotyped by PCR-restriction fragment length polymorphism (RFLP). The 182-bp PCR product containing Asn142Asp was digested with *MboI* restriction enzyme (New England BioLabs, Inc., Beverly, MA, USA), which cleaved the DNA fragment to 86- and 96-bp fragments if the polymorphism were present. Five per cent of those samples were further validated with DNA sequencing in a bidirectional manner.

### Statistical analysis

We performed an unpaired *t*-test was performed to calculate statistically significant differences between cases and controls in continuous independent variables (e.g., age, arsenic content in water, urine, nail, hair) by using GraphPad InStat Software (Graphpad Software Inc., San Diego, CA, USA). A chi-square test was used to compare the distribution of sex and haplotypes between two groups. The risk of developing an arsenic-induced skin lesion was calculated as the odds ratio (OR) with a 95% confidence interval (CI), as well as the *p*-value for exonic SNPs identified in *PNP*, *As3MT*, *GSTO1,* and *GSTO2*. Extended haplotypes were calculated using the ARLEQUIN software package (Schneider et al. 2000), and linkage disequilibrium (LD; *r*^2^) was calculated using the Haploview software package, version 3.32 (Barrett 2005). We performed a chi-square test using proper contingency tables to check the overall variation of the haplotypes between cases and controls, followed by association of a specific haplotype (having all three minor alleles) against the combined haplotypes with arsenicism.

## Results

Descriptive characteristics of the total study population are summarized in Table 1. Age, sex, and sociodemographic characteristics are similar in case and control groups. With regard to addiction status (both tobacco smoking and chewing), we did not find any significant deviation between two study groups (*p* = 0.37), even after stratifying the population in males (*p* = 0.76) and females (*p* = 0.66). The difference in mean arsenic content in drinking water and urine between the two groups was not significant (*p* = 0.92 and *p* = 0.64). When arsenic content in nail and hair was compared, it was much higher in cases (*p* < 0.001 and *p* < 0.001, respectively) compared with that in the control group.

We screened the entire exonic portion of the four genes in 100 chromosomes (25 cases and 25 controls) to get an initial overview of the commonly occurring SNPs in our population. The minor allele frequencies (MAF) of each variant were also calculated [Supplemental Material, Table 2 (online at http://www.ehponline.org/members/2008/10581/suppl.pdf)]. We detected eight commonly occurring polymorphisms (His20His, Gly51Ser, Pro57Pro in *PNP*; Met287Thr in *As3MT*; Ala140Asp, −/AGG, Glu208Lys in *GSTO1*; and Asn142Asp in *GSTO2*) in this population, but other reported polymorphisms (rs17881206 at 5′ UTR, rs7785 at 3′ UTR, Ala174Ala in *PNP*; Arg173Trp, Thr306Ile, Ile132Phe, Tyr135Asn, Gly140Ala in *As3MT*; Ser86Cys, Thr217Asn, Ala236Val in *GSTO1*; and Val114Ile, Leu158Ile, Cys130Tyr in *GSTO2*) were monomorphic in our population. We identified some novel SNPs in *PNP* and *GSTO1*. We studied genotypic distribution for these eight commonly occurring SNPs in 229 cases and 199 controls and estimated the association of each of these polymorphisms with the risk of developing arsenic-induced skin lesions (Table 2). The OR between case and control groups was calculated designating the major allele in the homozygous state as referent genotype for all the SNPs. Results indicated that only *PNP* polymorphisms were associated with arsenicism. Genotypes carrying at least one minor allele (i.e., heterozygous and homozygous genotype for minor alleles) for all three *PNP* polymorphisms were significantly overrepresented in cases [OR = 1.69 (95% CI, 1.08–2.66) for codon 20; OR = 1.66 (95% CI, 1.04–2.64) for codon 51; and OR = 1.67 (95% CI,1.05–2.66) for codon 57]. The results thus suggest that these individuals carrying minor alleles, either in heterozygous or homozygous condition, are at risk of developing arsenic-induced skin lesions.

Pairwise haplotypes of *PNP* polymorphisms and their corresponding LD measured as *r*^2^ values were estimated. The LD values were significant among all three paired loci in both the study groups (0.85 for codons 20 and 57; 0.85 for codons 20 and 51; and 0.98 for codons 51 and 57) (not shown). Extended haplotypes considering all three *PNP* loci were constructed in the following order: codon 20–codon 51–codon 57 (Table 3). It is evident from the haplotypic distribution that the haplotype having highest frequency is the one that contains the major alleles at the respective loci, that is, C-G-C, followed by T-A-T, T-G-C, and C-A-T. The other three haplotypes found showed distribution exclusive to either case or control. Overall, there was no significant variation of haplotype frequency between cases and controls (*p* = 0.17). However, the specific T-A-T haplotype (i.e., having the minor alleles in the respective loci), against all other haplotypes combined, was significantly associated with arsenicism (*p* = 0.048) (Table 3).

## Discussion

Understanding the detailed metabolic pathway of ingested arsenic is critical in defining the molecular mechanisms of arsenic toxicity and carcinogenicity. The toxic or carcinogenic potential of ingested arsenic depends on a subtle balance between various arsenical intermediates because of retention or excretion. In the present study, the intake and excretion of arsenic between cases and controls were similar, but cases showed significantly higher arsenic accumulation in nail and hair. This load, coupled with the genetic idiosyncrasies of the arsenic-metabolizing genes, determines whether a particular individual will be susceptible to arsenic toxicity. This study shows that of eight exonic polymorphisms studied in four arsenic-metabolizing genes, three polymorphisms of *PNP* were significantly associated with arsenicism. The following section is a detailed account of the exonic SNPs identified in our population, with special emphasis on their distribution bias among the cases and controls, if any.

### PNP

The Gly51Ser polymorphism at codon 51 resides in the proximity of the phosphate or arsenate-binding region of PNP enzyme (residues 56–69). Therefore, the substitution at this position may create some structural modification affecting protein function. However, a functional assay with variants in a case with severe combined immunodeficiency syndrome (Aust et al. 1992; Williams et al. 1987) showed no drastic alteration in enzyme activity. However, the possibility of a conservative change of glycine (nonpolar, uncharged) to serine (polar) to modulate the susceptibility level of individuals exposed to this xenobiotic cannot be ruled out without proper functional assays with the arsenic-exposed individuals. Consistent with the findings of Yu et al. (2003), the strong LD (*r*^2^ > 80) between codon 20 and codon 57 loci with codon 51 (Gly51Ser) perhaps explains the association of these two synonymous SNPs with arsenicism. Meza et al. (2005), in his studies with the Mexican population, found no significant correlation between *PNP* polymorphisms and urinary arsenic metabolites.

### As3MT

A number of *in vivo* and *in vitro* studies established that As3MT enzyme is indispensable for conversion of the arsenic metabolites to their corresponding methylated products (Drobna et al. 2005, 2006). In our study, screening of the entire coding region revealed only one exonic SNP (Met287Thr) in our population, with a heterozygosity of about 9%, similar to studies with African-American and white American populations (Wood et al. 2006). It should be pointed out that the human major allele at codon 287, *Met*, is actually the nonancestral allele and that 10 other primates studies encoded Thr at the corresponding position. It would be interesting to assess the basis of the allele flipping in the human and the functional alteration of the protein product with the variant allele, if any.

### GSTO1 *and* GSTO2

Six polymorphisms in the *GSTO1* gene (Ser86Cys, Ala140Asp, −/AGG, Glu208Lys, Thr217Asn, Ala236Val) have been reported in various ethnic groups of the world or cited in the databases. Our analysis of *GSTO1* could identify only three SNPs (Ala140Asp, −/AGG, Glu208Lys) among them. Generally, GSTO1 exhibits higher thiol transferase activity than GST. Tanaka-Kagawa et al. suggested that Ala140Asp polymorphism reduces thiol transferase activity of GSTO1 (Tanaka-Kagawa et al. 2003); however, another study found no such difference in enzyme activity due to the presence of the variant allele (Whitbread et al. 2003). A study in a Taiwanese population identified a significant association between the Asp140 variant of the GSTO1 gene variant and hepatocellular carcinoma, cholangio-carcinoma, and breast cancer (Marahatta et al. 2006). In fact, the frequency of the variant allele of the Asp140 variant of the GSTO1 gene varies significantly among world populations (Hirakawa et al. 2002; Whitbread et al. 2003). The frequency of the Asp140 variant in our population was 0.13, close to that of the Chinese population, reflecting a similarity within the Asian populations. Two other SNPs identified in our population (−/AGG and Glu208Lys) are reported to be associated with an inability to process inorganic arsenic. The −/AGG polymorphism is caused by a three-base deletion at the exon–intron border of exon 4, which might have potential for missplicing (Marnell et al. 2003). However, we could not find any significant difference in the genotypic distribution of any of the exonic SNPs between cases and controls.

There is 64% amino acid identity of GSTO2 enzyme with GSTO1. The minor allele frequency of the only exonic SNP identified in our population, Asn142Asp, is closely similar (0.28) to the European Australians in Canberra and the Chinese population from Hong Kong (Whitbread et al. 2003). This 142Asp variant is the major allele among Bantu Africans in Durban, Caucasian-American, Han-Chinese–American, and Mexican-American populations (Mukherjee et al. 2006), thus raising speculation about any ethnicity-specific selection bias. However, we found no association between the distribution of 142Asp variant and arsenicism in our population.

## Conclusion

This study highlights that genetic variants of PNP, one of the important enzymes in arsenic metabolism, are associated with development of arsenic-induced skin lesions. The study also provides basal knowledge of commonly occurring genetic polymorphisms in the protein-coding region of important arsenic-metabolizing genes. We did not find significant association of exonic SNPs in arsenic-metabolizing genes with arsenicism, except *PNP*. However, the interplay among the genes may be crucial in modulating the arsenic biotransformation pathway, and even minor changes in their functions can be reflected at phenotypic level and hence, in disease manifestation. Detailed functional studies are required in arsenic-exposed populations to understand the mechanisms involved. In this respect, gene knockouts can be used to examine the alterations in resulting in arsenic reduction and methylation pattern and the toxicologic consequences.

## Figures and Tables

**Table 1:** Descriptive characteristics of the study participants.

Parameter	Control [*n* (%)]	Case [*n* (%)]	*p*-Value
Total subjects	199	229	
Male	94 (47.24)	113 (49.34)	0.74
Female	105 (52.76)	116 (50.66)	
Age [years (mean ± SE)]	38.21 ± 0.95	39 ± 0.73	0.50
Addiction	80 (40.2)	103 (44.98)	0.37
Male			
Smoking (bidi/cigarette)	28 (50)	30 (43.48)	0.76
Tobacco chewing	14 (25)	19 (27.54)	
Both	14 (25)	20 (28.98)	
Female			
Smoking (bidi/cigarette)	6 (25)	7 (20.6)	0.66
Tobacco chewing	12 (50)	21 (61.8)	
Both	6 (25)	6 (17.6)	
Occupation			
Male			
Cultivation	42 (44.68)	68 (60.18)	
Business	14 (14.89)	16 (14.16)	
Daily wage earners	18 (19.15)	7 (6.19)	
Service	3 (3.19)	5 (4.42)	
Teacher	1 (1.06)	5 (4.42)	
Student	7 (7.45)	2 (1.77)	
Unemployed	9 (9.57)	10 (8.85)	
Female			
Housewife	90 (85.71)	99 (85.34)	
Cultivation	10 (9.52)	7 (6.03)	
Business	1 (0.95)	1 (0.86)	
Daily wage earners	2 (1.9)	4 (3.45)	
Service	1 (0.95)	2 (1.72)	
Student	1 (0.95)	1 (0.86)	
Unemployed	0	2 (1.72)	
Arsenic content (mean ± SE)			
Drinking water (μg/L)	161.71 ± 10.71	163.16 ± 10.00	0.92
Urine (μg/L)	283.28 ± 23.73	301.17 ± 28.68	0.64
Nail* (μg/g)	2.39 ± 0.13	5.39 ± 0.46	< 0.001
Hair* (μg/g)	1.61 ± 0.09	3.13 ± 0.24	< 0.001

* *p* < 0.001 [unpaired *t*-test (two-tailed)].

**Table 2:** Risk assessment of *PNP* polymorphisms between cases and controls.

Polymorphism	Control^a^ [*n* (%)]	Case^a^ [*n* (%)]	OR (95% CI)	*p*-Value
*PNP*				
C > T (codon 20)				
C/C	160 (80.4)	162 (70.7)	1.00 (referent)	0.02
C/T + T/T	39 (19.6)	67 (29.3)	1.69 (1.08–2.66)	
G > A (codon 51)				
G/G	154 (81.05)	160 (72.07)	1.00 (referent)	0.04
G/A + A/A	36 (18.95)	62 (27.93)	1.66 (1.04–2.64)	
C > T (codon 57)				
C/C	151 (80.75)	158 (71.5)	1.00 (referent)	0.04
C/T + T/T	36 (19.25)	63 (28.5)	1.67 (1.05–2.66)	
*As3MT*				
T > C (codon 287)				
T/T	181 (90.95)	206 (90.75)	1.00 (referent)	0.94
T/C + C/C	18 (9.05)	21 (9.25)	1.02 (0.53–1.98)	
*GSTO1*				
C > A (codon 140)				
C/C	161 (80.9)	171 (76.0)	1.00 (referent)	0.27
C/A + A/A	38 (19.1)	54 (24.0)	1.34 (0.84–2.13)	
-/AGG (Intron 4, splice donor site)				
AGG/AGG	188 (94.5)	211 (92.14)	1.00 (referent)	0.44
-/AGG	11 (5.5)	18 (7.86)	1.44 (0.67–3.17)	
G > A (codon 208)				
G/G	153 (76.9)	188 (82.1)	1.00 (referent)	0.23
G/A + A/A	46 (23.1)	41 (17.9)	0.72 (0.45–1.16)	
*GSTO2*				
A > G (codon 142)				
A/A	105 (52.8)	128 (55.9)	1.00 (referent)	0.58
A/G + G/G	94 (47.2)	101 (44.1)	0.88 (0.6–1.29)	

a Case, *n* = 229; *n* = control, 199. Discrepancy in the number of genotyped data in some of the loci is due to PCR failure even after multiple independent efforts.

**Table 3:** Extended haplotype frequencies of the study population.

	*PNP* haplotypes (codons 20, 51, 57)							
Population (*n*)	C-G-C	C-G-T	C-A-C	C-A-T	T-G-C	T-G-T	T-A-T^a^	*p*-Value
Control (187)	329	1	1	5	6	0	32	0.17
Case (221)	366	0	0	7	11	1	57	

a T-A-T haplotype was found to be overrepresented in cases (*p* = 0.048).
